# 

**Published:** 1918-02-23

**Authors:** 


### CONTENTS.

| | PAGE |
|---|---|
| **Some Medical Difficulties of Life Assurance** | 429 |
| **Temporary Hospital Staff Appointments** ... | 430 |
| **Hospital and Institutional News** ... ... ... | 431 |
| The Prince of Wales and Limbless Sailors and Soldiers—*The Modern Hospital*—A Steady Development—The New Chief of the Pavilion Hospital, Brighton—Institutions and the New Rations—The Endowment of Research—The Release of Medical Students—The Year's Work at Cardiff—What Remains to be Done—Extension Problems: Two Points of View—Women Collectors at Gravesend—The First Annual Reports—The Disabled Men's View of Training—Treatment of Tuberculosis Patients—A Generous Provincial President—The Problem of the Mentally Defective—The Tuberculosis Trust in Scotland—St. Thomas's Hospital Speech Clinic—This Week's Drug Market. | |
| **Lord Lister** ... ... ... ... ... ... ... | 435 |
| V. His Early Researches and Conclusions. | |
| **The Great War** ... ... ... ... ... ... | 437 |
| II. With the Wounded at the Front. | |
| **Dr. Henry Maudsley** ... ... ... ... ... | 438 |
| **The Board of Control** ... ... ... ... | 439 |
| Lunacy in 1916. | |
| **The Record of the Red Cross** ... ... ... | 440 |
| The Temptation of Economical Management. | |
| **Annual Meetings** ... ... ... ... ... ... | 440 |
| **The Matrons' and Sisters' Department** ... | 441 |
| The Growth of the Nursing Profession: IV. Some Economic and Practical Aspects. | |
| Round the Hospitals. | |
| His Majesty's Gift to the Nation's Fund—Miss Ray, R.R.C.—Her Great Work at King's—Sound Finance of the College—Nurses' Registration Fees Paid and Banked—Nurses Awaiting the *Amende Honorable.* | |
| **Food in War-Time** ... ... ... ... ... ... | 445 |
| The Importance of the Vegetable Course. | |
| **Parliament and the Profession** ... ... ... | 445 |
| **Addenbrooke's Hospital, Cambridge: Quarterly Court** ... ... ... ... ... ... ... ... | 446 |
| **Increased Capitation Grants to Civil Hospitals** | 446 |
| **The Editor's Letter-Box** ... ... ... ... | 447 |
| The Shortage of Husbands—Increase in War Office Capitation Grant—"Words and Things"—The First Annual Report. | |
| **Leicester Saturday Hospital Society** ... ... | 448 |
| **Public Pharmacists' Association** ... ... ... | 448 |
| **Matrons' and Sisters' Appointments** ... ... | 448 |

